# Maternal risk factors and neonatal outcomes associated with low birth weight

**DOI:** 10.3389/fgene.2022.1019321

**Published:** 2022-09-28

**Authors:** Yang Shaohua, Zheng Bin, Liu Mei, Zhai Jingfei, Qiao Pingping, He Yanping, Zhu Liping, Yan Jiexin, Mao Guoshun

**Affiliations:** ^1^ Wenzhou Medical University, Wenzhou, Zhejiang, China; ^2^ Fuyang People’s Hospital Pediatrics, Fuyang, China

**Keywords:** low birth weight, risk factors, marital status, gestational age, incidence

## Abstract

This study aims to evaluate the incidence of low birth weight (LBW) and related maternal risk factors (during pregnancy or childbirth) and neonatal outcomes. A retrospective cross-sectional study design was used to select 7,421 pregnant women who gave birth in our hospital from January 2018 to June 2021. The data were analyzed using STATA 14.1, and the dependent variable (LBW) and risk were analyzed by the chi-square test of independence. The association between factors is used to determine the factors related to LBW through bivariate and multivariate logistic regression. The incidence of LBW in this study was 4.77%. Compared with single pregnant women, the probability of newborn LBW in married pregnant women is 40% lower (AOR = 0.60 95%CI: 0.40–0.90, *p* = 0.013). Compared with gestational age less than 37 weeks, the LBW probability of gestational age 37–42 and 42 weeks or older is 85 and 81% lower respectively (AOR = 0.15 95% CI: 0.10–0.24, *p* = 0.001; AOR = 0.19 95 %CI: 0.09–38, *p* = 0.001), compared with normal pregnant women, the probability of neonatal LBW among pregnant women with hypertension is 94% higher [AOR = 1.94 (95% CI: 1.39–2.74, *p* = 0.001). Compared with neonates with normal birth weight, neonates with LBW are at Apgar 1 min And Apgar 5 min score is lower than 7 (AOR = 0.52 95%CI: 0.37–0.73, *p* = 0.001, AOR = 0.54 95%CI: 0.38–0.75, *p* = 0.001) higher risk. In conclusion, women’s marital status (single), gestational age (<37 weeks), and combined hypertension are independently associated with LBW, and the higher risk of Apgar 1 min and Apgar 5 min scores <7 is an independent result of LBW.

## Introduction

Birth weight is critical for neonatal survival, physical health, and development (Sharma et al., 2015). Low birth weight (LBW), defined as birth weight below 2,500 g, is considered a major health problem for newborns (World Health Organization, 2014). It is the leading cause of 40%–60% of neonatal deaths worldwide (UNICEF and United Nations Childr en’s Fund (UNICEF), 2008). Currently, the incidence of LBW worldwide is approximately 20 percent, of which 95 percent occur in low- and middle-income countries (World Health Organization, 2014). The incidence of LBW has not declined in any way over the past decade, which is of concern to medical staff, policymakers, and researchers (World Health Organization, 2014). Babies born with LBW are at risk for many health problems, including: hypothermia, hypoglycemia, cognitive impairment, malnutrition, etc (Qian and Gu, 2020; Shandong Province Multi-center Prognosis Evaluation Cooperative Group for Very Low Birth Weight Infants, 2020). In addition, LBW infants are at risk of Complication mortality is 20 times higher (Sharma et al., 2015).

The incidence of LBW continues to increase year by year despite efforts and a number of strategies specified (World Health Organization, 2014). The UNICEF-WHO 2019 report shows that global progress in reducing the incidence of LBW was slower in the period 2010–2015 compared to the period 2000–2009, thereby impacting the prevention of neonatal mortality and reducing stunting and the number of wasted children (World Health Organization, 2019). Understanding the incidence and influencing factors of LBW births is important for medical staff, hospital administrators, and policy makers because the feedback gathered can be used to implement the right strategies related to LBW prevention/reduction. Therefore, this study aimed to assess the incidence of LBW and associated maternal risk factors (during pregnancy or delivery) and neonatal outcomes.

## Methods

### Study population

This study adopted a retrospective cross-sectional study design, and selected 7,421 pregnant women who delivered in our hospital from September 2018 to June 2021 for retrospective analysis. Inclusion criteria: singleton pregnancy; neonates without congenital diseases. Exclusion criteria: incomplete maternal and infant information, unknown neonatal status (i.e., dead or alive), stillbirth, missing birth weight information, non-singleton pregnancy. The study was approved by the hospital ethics approval committee (Ethical approval K20180412), but no informed consent was obtained because it was a retrospective study.

### Data collection

Based on a review of relevant literature (Agbozo et al., 2016; Mohammed et al., 2019), a structured data extraction questionnaire was designed and data was collected through the hospital electronic information recording system. The data was divided into two parts: sociodemographic characteristics of pregnant women and newborns, and obstetric characteristics. Sociodemographic characteristics include: age, marital status, education level, occupation of pregnant women. Obstetric characteristics were as follows: number of pregnancies, fetal presentation, gestational age, mode of delivery, hypertensive disorders, bleeding disorders, UTI/STI, premature rupture of membranes (PROM), and preeclampsia, and neonatal factors included: Neonatal gender, Apgar 1 min score, Apgar 5 min score, neonatal resuscitation, crying at birth.

### Data analysis

Data were analyzed by STATA 14.1, descriptive statistics were used for categorical data, chi-square test was used to test the association between the dependent variable (LBW) and LBW risk factors, and bivariate and multivariate logistic regression was used to identify factors associated with LBW, significant variables in bivariate logistic regression were used for multivariate logistic regression and *p* < 0.05 values were considered statistically significant.

## Result

### Sociodemographic characteristics

346 (4.78%) newborns weighed less than 2,500 g, most (58.2%) pregnant women were between 21–30 years old, 83.6% were married, the majority (63.0%) of pregnant women had secondary or higher education, and male newborns (50.9%) were slightly higher than females (50.9%, Table 1).

**TABLE 1:** Maternal sociodemographic factors associated with low birth weight.

		LBW(*N* = 346)	NBW (*N* = 7,075)	Total (*N* = 7,421)	χ2	*p*	COR [95%CI]	*p*
Age	≤20	19 (5.49)	424 (5.99)	443 (5.97)			1	
	21–30	188 (54.3)	4,135 (58.4)	4,323 (58.3)			0.61 [0.34–1.07]	0.087
	31–40	132 (38.2)	2,421 (34.2)	2,553 (34.4)			0.58 [0.32–1.05]	0.070
	>40	7 (2.02)	95 (1.34)	102 (1.37)	3.80	0.284	0.89 [0.24–3.24]	0.858
Marital status	Divorced/widowed	91 (26.3)	1,038 (14.7)	1,129 (15.2)			1	
	Married	247 (71.4)	5,967 (84.3)	6,214 (83.7)			0.69 [0.47–1.00]	0.043
	Single	8 (2.31)	168 (2.37)	176 (2.37)	4.07	0.130	0.47 [0.05–4.14]	0.497
Education level	Medium and above	221 (63.9)	4,451 (62.9)	4,672 (63.0)			1	
	Elementary and below	125 (36.1)	2,624 (37.1)	2,749 (37.0)	1.59	0.207	0.74 [0.46–1.18]	0.208
Profession	Civil servant	62 (17.9)	1,347 (19.4)	1,409 (19.0)			1	
	Housewife	93 (26.9)	1,613 (22.8)	1706 (23.0)			1.40 [0.84–2.33]	0.191
	Business	128 (37.0)	2,858 (40.4)	2,986 (40.2)			1.14 [0.76–1.70]	0.525
	Student	63 (18.2)	1,254 (17.7)	1,317 (17.7)	1.80	0.614	1.22 [0.65–2.28]	0.538

### Maternal sociodemographic factors associated with low birth weight

**FIGURE 1:**
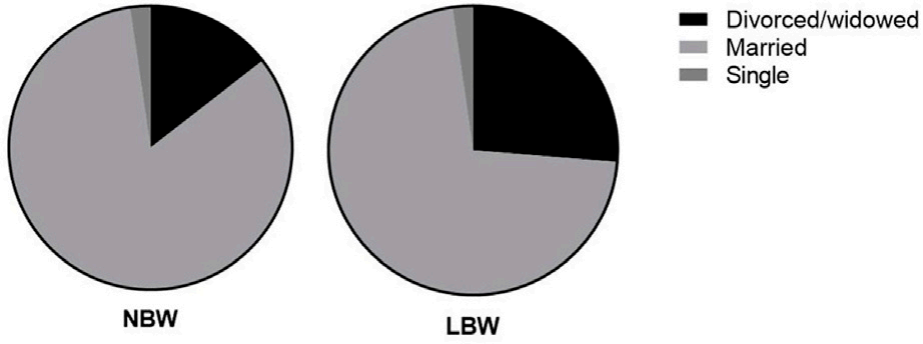
Marital status of the two groups.

Bivariate logistic regression analysis showed that compared with single pregnant women, married pregnant women were 31% less likely to have low birth weight neonates [COR = 0.69, 95%CI: 0.47–1.00, *p* = 0.043, Table 1; Figure 1).

### Antenatal and obstetric factors associated with low birth weight

**FIGURE 2:**
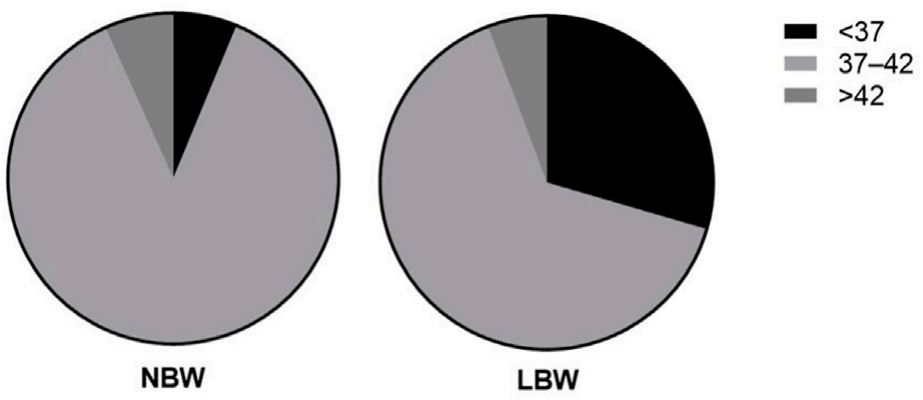
Gestational age status of the two groups.

**FIGURE 3:**
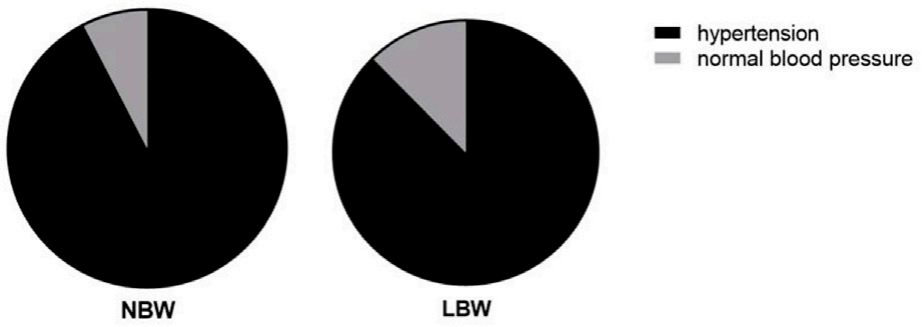
Blood pressure status of the two groups of people.

Chi-square test of independence showed that gestational age (χ2 = 102.0, *p* = 0.001, Figure 2) and hypertension (χ2 = 5.76, *p* = 0.016, Figure 3) were significantly associated with LBW. Bivariate logistic regression analysis further showed that compared with gestational age less than 37 weeks, the incidence of LBW was reduced by 85% (COR = 0.15 95%CI: 0.10–0.22) at gestational age of 37–42 weeks, and the incidence of LBW was reduced by 83% at gestational age of more than 42 weeks (COR = 0.17 95%CI: 0.08–0.34, *p* < 0.001), the prevalence of LBW was 76% higher among pregnant women with hypertensive disorders (COR = 1.76 95%CI: 1.10–2.81, *p* = 0.018, Table 2).

**TABLE 2:** Antenatal and obstetric factors associated with low birth weight.

		LBW (*N* = 346)	NBW (*N* = 7,075)	Total (*N* = 7,421)	χ2	*p*	COR [95%CI]	*p*
Number of pregnancies	1	204 (59.0)	67 (27.8)	4,307 (25.9)			1	
	2	118 (34.1)	68 (28.2)	2,672 (28.7)			0.89 [0.60–1.31]	0.549
	≥3	24 (6.94)	45 (18.7)	442 (20.2)			0.82 [0.53–1.27]	0.376
Gender	male	178 (51.4)	3,601 (50.9)	3,779 (50.9)			1	
	Female	168 (48.6)	3,474 (49.1)	3,642 (49.1)			0.93 [0.65–1.34]	0.712
Gestational age	<37	102 (29.5)	443 (6.26)	545 (7.34)			1	
	37–42	224 (64.7)	6,153 (87.0)	6,377 (85.9)			0.15 [0.10–0.22]	0.001
	>42	20 (5.78)	479 (6.77)	499 (6.72)	102.0	0.001	0.17 [0.08–0.34]	0.001
Fetal presentation	head presentation	307 (88.7)	6,290 (88.9)	6,597 (88.9)			1	
	breech presentation	36 (10.4)	724 (10.2)	760 (10.3)			1.23 [0.64–2.37]	0.542
	shoulder presentation	3 (0.87)	61 (0.86)	64 (0.86)			1.28 [0.25–6.66]	0.766
Mode of delivery	SVD	221 (63.9)	4,376 (61.9)	4,597 (61.9)			1	
	CS	125 (36.1)	2,699 (38.1)	2,824 (38.1)			1.14 [0.85–1.53]	0.366
CS-type	Involuntary CS	71 (20.5)	1,681 (23.8)	1752 (23.6)			1	
	Voluntary CS	54 (15.6)	1,018 (14.4)	1,072 (14.5)	1.32	0.250	0.75 [0.45–1.23]	0.251
Hypertension	no	301 (87.6)	6,537 (92.4)	6,838 (92.1)			1	
	Yes	45 (12.4)	538 (7.60)	583 (7.86)	5.76	0.016	1.76 [1.10–2.81]	0.018
Antepartum hemorrhage	no	287 (82.9)	5,802 (82.0)	6,089 (82.1)			1	
	Yes	59 (17.1)	1,273 (18.0)	1,332 (17.9)	0.26	0.607	0.91 [0.62–1.32]	0.607
UTI/STI	no	332 (96.0)	6,813 (96.3)	7,145 (96.3)			1	
	Yes	14 (4.05)	262 (3.70)	276 (3.72)	0.46	0.500	1.28 [0.63–2.61]	0.501
PROM	no	302 (87.2)	6,297 (89.0)	6,599 (88.9)			1	
	Yes	44 (12.7)	778 (11.0)	822 (11.1)	0.01	0.910	0.98 [0.63–1.50]	0.910
Preeclampsia	no	272 (78.6)	5,720 (80.8)	5,992 (80.7)			1	
	Yes	74 (21.4)	1,355 (19.2)	1,429 (19.3)	0.04	0.837	1.04 [0.73–1.47]	0.837

### Estimating risk of neonatal outcomes based on birth weight

Chi-square test of independence showed that LBW and Apgar 1 min score <7 (χ2 = 27.06, *p* = 0.001), Apgar 5 min score <7 (χ2 = 21.81, *p* = 0.001), and neonatal resuscitation (χ2 = 5.12, *p* = 0.024) was associated with crying at birth (χ2 = 7.11, *p* = 0.008). Bivariate logistic regression analysis also showed that LBW neonates had a higher risk of Apgar 1 min and Apgar 5 min scores less than 7 and a 52% higher neonatal resuscitation rate compared with normal-weight neonates (COR = 1.52 95%CI:1.06–2.19, *p* = 0.024) and a 40% lower rate of postnatal crying (COR = 0.60 95%CI: 0.60–0.87, *p* = 0.008, Table 3).

**TABLE 3:** Estimated risk of neonatal outcomes based on birth weight.

		LBW (*N* = 346)	NBW (*N* = 7,075)	Total (*N* = 7,421)	χ2	*p*	COR [95%CI]	*p*
Apgar 1 min score	<7	152 (43.9)	1907 (27.0)	2059 (27.7)			1	
	≥7	194 (56.1)	5,168 (73.0)	5,362 (72.3)	27.06	0.001	046 [0.34–0.61]	0.001
Apgar 5 min score	<7	79 (22.8)	849 (12.0)	928 (12.5)			1	
	≥7	267 (77.2)	6,226 (88.0)	6,493 (87.5)	21.81	0.001	0.42 [0.29–0.61]	0.001
Neonatal resuscitation	no	273 (78.9)	5,943 (84.0)	6,216 (83.8)			1	
	Yes	73 (21.1)	1,132 (16.0)	1,205 (16.2)	5.12	0.024	1.52 [1.06–2.19]	0.024
Cry after birth	no	70 (20.2)	847 (11.1)	917 (12.4)			1	
	Yes	276 (79.8)	6,228 (88.9)	6,504 (87.6)	7.11	0.008	0.60 [0.41–0.87]	0.008

### Multivariate logistic regression showing factors associated with low birth weight

Married pregnant women had a 40% lower chance of neonatal LBW compared with single pregnant women (AOR = 0.60 95%CI: 0.40–0.90, *p* = 0.013), compared with less than 37 weeks gestational age, the odds of LBW were 85 and 81% lower for gestational age 37–42 weeks and over 42 weeks (AOR = 0.15 95%CI: 0.10–0.24, *p* = 0.001; AOR = 0.19 95%CI: 0.09–0.38, *p* = 0.001), compared with normal pregnant women, pregnant women with hypertensive disorders had a 94% higher chance of neonatal LBW (AOR = 1.94 95%CI: 1.39–2.74, *p* = 0.001), compared with neonates with normal birth weight, LBW neonates had Apgar 1min and Apgar 5 min scores of less than 7 (AOR = 0.52 95%CI: 0.37–0.73, *p* = 0.001, AOR = 0.54 95%CI: 0.38–0.75, *p* = 0.001, Table 4) risk was higher.

**TABLE 4:** Multivariate logistic regression showing factors associated with low birth weight.

		AOR	95%CI	*p*
Marital status	single		1	
	Married	0.60	0.40–0.90	0.013
	divorced/widowed	0.46	0.05–4.15	0.487
Gestational age	<37		1	
	37–42	0.15	0.10–0.24	0.001
	>42	0.19	0.09–0.38	0.001
	no		1	
	Yes	1.42	0.85–2.38	0.182
Hypertension	no		1	
	Yes	1.94	1.39–2.74	0.001
Apgar 1 min score	<7		1	
	≥7	0.52	0.37–0.73	0.001
Apgar 5 min score	<7		1	
	≥7	0.54	0.38–0.75	0.001
Neonatal resuscitation	no		1	
	Yes	0.61	0.34–1.10	0.100
Cry after birth	no		1	
	Yes	0.65	0.36–1.19	0.162

## Discussion

Birth weight is critical for neonatal survival, physical health, and development, so regional assessments of the incidence of LBW and its specific causes are recommended to alert healthcare workers and policy makers to appropriate interventions to improve women’s health before, during and after pregnancy. A total of 7,421 mother-infant pairs were included in this study, and the incidence of neonatal LBW was 4.77%. Our findings are lower than the 5.3% reported in Hu Meina’s article “Low Birth Weight Incidence and Influencing Factors”, and higher than the 4.3% reported in Wan Lixin’s “2018 Neonatal Low Birth Weight Incidence and Influencing Factors Analysis in Jilin Province” (Hu et al., 2019; Wan et al., 2020), and the difference in prevalence in different regions may be due to different living habits, living environment and economic factors.

The present study found a positive association between marital status and LBW in a logistic regression model, with married women having a 40% lower chance of having a newborn with LBW. This finding is consistent with previous studies reporting that pregnant women are married as a preventive factor for LBW (Agorinya et al., 2018). This is because most men (husbands) are the head of the household and have an important role in meeting the needs of their wives, including the decision to seek skilled nursing services during pregnancy. The lower odds of LBW newborns among married women may be due to the financial, mental, and physical support of the husband to keep the woman in a good state of mind during pregnancy and childbirth. This study observed a significant association between gestational age and LBW. Neonates born at term (37 weeks gestation and older) were 85% less likely to develop LBW compared with preterm infants (AOR = 0.15 95%CI: 0.10–0.24, *p* = 0.001). The results of the current study are similar to those reported in the previous literature (Liu et al., 2021). It was further shown that neonates born before 37 weeks of gestational age were more likely to develop LBW than neonates born at term. Furthermore, the present findings are consistent with another previously conducted study that indicated that gestational age was independently associated with the incidence of LBW: neonates born at 37 weeks of gestation or older were protective against LBW (Gebremedhin et al., 2015; Mengesha et al., 2017). It is clear that if the gestational age of the fetus is below the normal time frame (37 weeks gestation), there will be a dramatic loss of fetal weight due to preterm birth (World Health Organization, 2017). Therefore, in order to prevent preterm birth, any gynecological, medical or other conditions that may lead to preterm birth during pregnancy must be addressed promptly.

The current study found a clear association between hypertensive disorders of pregnancy (HDP) and LBW. Multiple studies worldwide have reported that women with HDP have a higher incidence of LBW than women with normotensive blood pressure (Wang et al., 2016; Zhang et al., 2016; Shen et al., 2019). Their findings are consistent with a secondary analytical survey conducted by WHO in low- and middle-income countries, confirming that women with HDP have twice the risk of having LBW babies (Adu-Bonsaffoh et al., 2017). *In vitro* experiments have confirmed that in gestational hypertension, trophoblast invasion of the spiral arteries supplying the placenta is reduced, in addition, due to reduced uteroplacental blood flow, resulting in small gestational age, preterm birth and intrauterine growth restriction, thus predisposing newborns to suffering from LBW (de Souza Rugolo et al., 2011; Amaral et al., 2017). Therefore, prompt and effective care for women with HDP is essential to reduce their complications and LBW. LBW infants were at higher risk for lower Apgar 1 min and Apgar 5 min scores than infants with normal birth weight. Likewise, previous studies have reported that infants born with LBW have an increased risk of low Apgar scores at the first and fifth minutes (Mitao et al., 2016). A study by Coutinho et al. (2018) found a significant association between neonates with LBW at birth and low Apgar scores at the first and fifth minutes. The similarity of the findings confirms that the incidence of low Apgar scores is inversely related to birth weight.

In conclusion, this study revealed a low incidence of LBW (4.77%), and that women’s marital status (single pregnant women), gestational age (<37 weeks), and comorbid hypertensive disorders were independent risk factors associated with LBW, while in Apgar Higher risk of 1 min and Apgar 5 min scores less than seven was an independent outcome of low birth weight. The current findings contribute to an understanding of the incidence of LBW and its influencing factors, which can guide healthcare providers, hospital administrators, and planners in the development and implementation of appropriate clinical and public health strategies aimed at reducing LBW. However, this study still has certain limitations. For example, although the sample size investigated is rich, most of them are from a single region, and the reference significance for the whole country is still limited. Secondly, the clinical factors included in this study are relatively few, and further research is needed supplement.

## Data Availability

The original contributions presented in the study are included in the article/supplementary material, further inquiries can be directed to the corresponding authors.
